# Asparagine Synthetase-Mediated l-Asparagine Metabolism Disorder Promotes the Perineural Invasion of Oral Squamous Cell Carcinoma

**DOI:** 10.3389/fonc.2021.637226

**Published:** 2021-03-10

**Authors:** Yong Fu, Liang Ding, Xihu Yang, Zhuang Ding, Xiaofeng Huang, Lei Zhang, Sheng Chen, Qingang Hu, Yanhong Ni

**Affiliations:** ^1^ Central Laboratory of Stomatology, Nanjing Stomatological Hospital, Medical School of Nanjing University, Nanjing, China; ^2^ Department of Oral and Maxillofacial Surgery, Nanjing Stomatological Hospital, Medical School of Nanjing University, Nanjing, China; ^3^ Department of Oral and Maxillofacial Surgery, Affiliated Hospital of Jiangsu University, Zhenjiang, China; ^4^ Department of Oral Pathology, Nanjing Stomatological Hospital, Medical School of Nanjing University, Nanjing, China

**Keywords:** amino acids metabolism, asparagine synthetase (ASNS), l-asparagine, perineural invasion (PNI), oral squamous cell carcinoma (OSCC)

## Abstract

Dysregulated amino acids metabolism reciprocally interplays with evolutionary phenotypic characteristics of cancer cells to enhance metastasis. The high metastasis potential of oral squamous cell carcinoma (OSCC) can manifest with perineural invasion (PNI). We here aimed to determine the role of amino acids metabolism in OSCCs with different PNI statuses. Targeted metabolomics was used to quantify 48 amino acids in 20 fresh OSCC samples and 25 amino acids were successfully detected, within which 9 were significantly up-regulated in PNI positive (PNI^+^) samples. As its highest area under the curve value (0.9063), l-asparagine was selected as the biomarker to distinguish PNI^+^ from PNI negative (PNI^−^). Then, the key enzyme of l-asparagine, asparagine synthetase (ASNS), was investigated using immunohistochemistry with 86 OSCC patients. The results showed that ASNS mainly expressed in tumor epitheliums and positively correlated with lymph node metastasis and PNI. Moreover, subgroup survival analysis revealed that ASNS expression combined with PNI status significantly improved their prognostic value, which was confirmed by the TCGA OSCC cohort (n = 279). To validate whether ASNS promotes PNI, we determined ASNS expression levels in five OSCC cell lines and one normal oral keratinocyte, and HSC3 showed the lowest ASNS level but CAL33 had the highest. Therefore, HSC3 and CAL33 (or PBS as control) were selected and injected separately into sciatic nerves to construct the *in vivo* PNI mouse models. Although both models eventually developed the hind-limb paralysis, nerve dysfunction in the CAL33 model progressed significantly earlier than HSC3 (Day 9 vs. Day 24). Besides, CAL33 migrated significantly farther than HSC3 in the nerve microenvironment (*P* = 0.0003), indicating high ASNS expression is indispensable for OSCC progression, especially PNI formation, through l-asparagine metabolism alteration. This study provides novel insights into how amino acids metabolism disorders alter tumor neurotropism which helps cancer metastasis.

## Introduction

Lip and oral cavity cancers are ranked among the top 15 most common cancers in the world, accounting for 500 550 cases out of which 177 384 patients succumbed (1). As the most common type of oral cancer, the 5-year overall survival rate of treated patients with oral squamous cell carcinoma (OSCC) remains approximately 60% because of its highly invasive and metastatic potential even at the early stage (2–4). Perineural invasion (PNI), as one of the significant oncologic features, has been strongly associated with the aggressive behavior leading to a poor prognosis (5).

PNI, the process of neoplastic invasion of nerves, also has been called neurotropic carcinomatous spread and perineural spread. The definition of PNI that widely accepted is that tumor cells are in close proximity to a nerve involving at least 33% of its circumference or within any of the 3 layers (the epineurium, perineurium and the endoneurium) of the nerve sheath (6). Different phenotypes meeting the current criteria of PNI have been well illustrated: Tumor cells inside nerve sheaths; Tumor cells surrounding at least 33% of the nerve circumference, thus the prevalence of PNI in OSCC was reported up to 82% (7).

In addition to OSCC, PNI indicates poor prognosis in various solid cancers, such as pancreatic ductal adenocarcinoma, prostate cancer, gastric carcinoma and cervical cancer et al., but the mechanism behind is still unclear (8). For example, Schwann cells the important components of peripheral nerves were shown to promote cancer dispersion along nerves through direct contact with cancer cells (9). Furthermore, neurotrophins and their receptors, chemokines, and matrix metalloproteinase have been demonstrated as the molecular mechanism driving PNI (10–14). However, few studies have focused on the metabolism dysregulation behind PNI.

Metabolic reprogramming has been shown to be an important hallmark of cancers. Most of the metabolomics studies in oral cancers focus on the metabolic profiles of saliva, serum, and tumor tissues in order to identify potential biomarkers for screening and early diagnosis (15). In addition to increased glucose and fatty acids metabolism during cancer progression, amino acid metabolism also increases to match demands for cancer cells growth and metastasis (16). For example, by a combination of non-targeted and targeted metabolomics, a panel including three amino acids (l-glutamate, l-aspartic acid, and l-proline) was identified as potential diagnostic biomarkers of OSCC (17). l-tryptophan metabolism promotes tumor invasion, metastasis and dysregulates immune cells infiltration thereby accelerating cancer progression (18, 19). Moreover, amino acids metabolism was also distinct at different distances from surgical margins in OSCC (20). Here, we hoped to further elucidate the amino acid metabolism alteration behind different PNI statuses.

In this study, 20 prospectively collected primary OSCC tissues were used for quantification of 48 amino acids using ultra-high-performance liquid chromatography-tandem mass spectrometer (UHPLC-MS/MS). After the differential amino acids analysis, the metabolite with the highest area under the curve (AUC) value to distinguish different PNI statuses was selected and its key enzyme were evaluated for its clinical value. Meanwhile, a mouse model was successfully constructed by injecting cancer cells into the sciatic nerves to study PNI *in vivo*. The hypothesis for this study is that dysregulated amino acids metabolism affects PNI and ultimately promotes OSCC progression.

## Materials and Methods

### Patients and Tissue Samples

**Figure 1:**
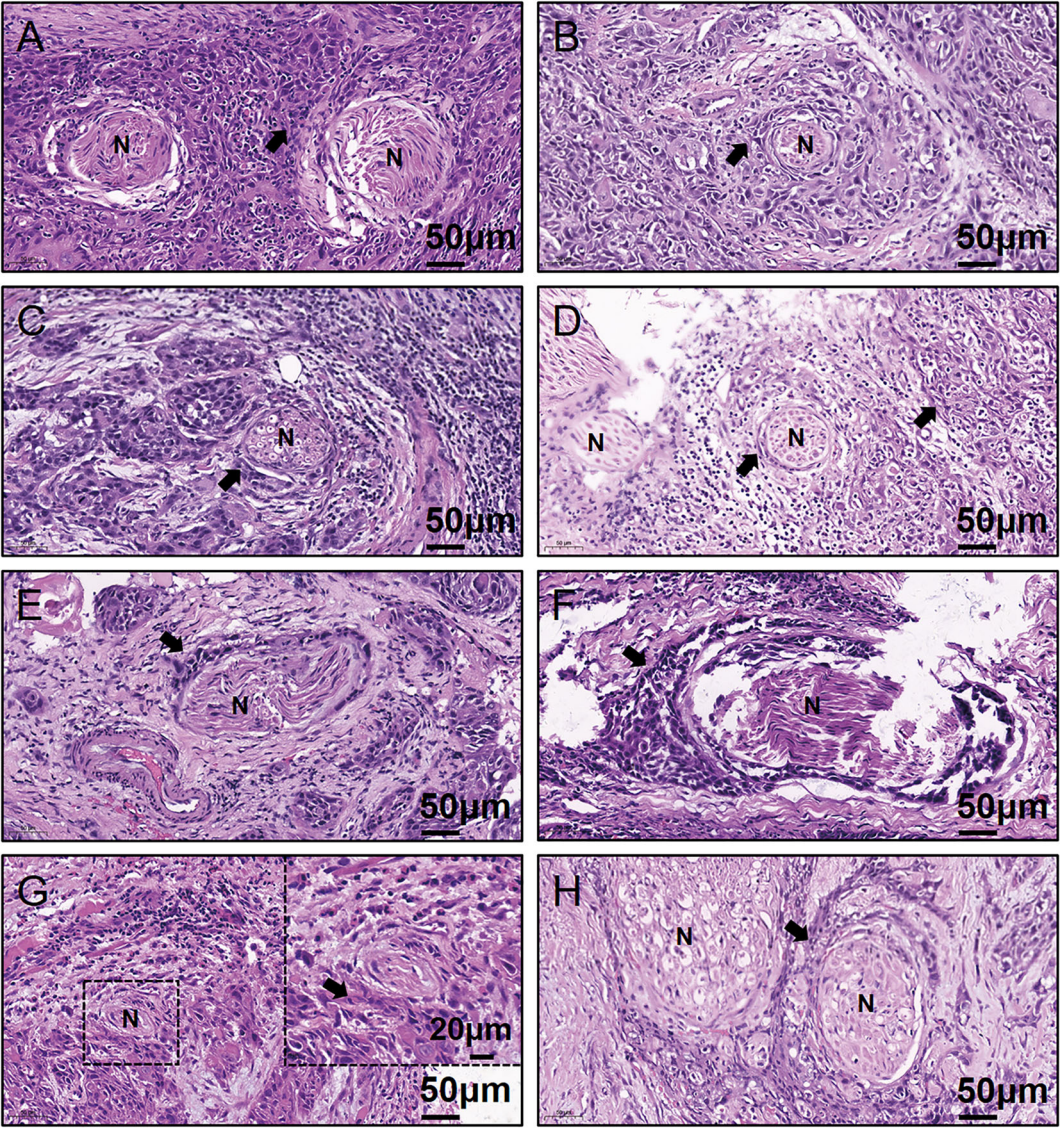
Representative H&E-stained images for the eight PNI^+^ OSCC samples. **(A–H)** Patient #13 to #20. N, Nerve; H&E, hematoxylin & eosin; PNI, perineural invasion; OSCC, oral squamous cell carcinoma; +, positive; The black arrow indicates the tumor.

Written informed consent for participation, including use of tissue samples, was obtained from each patient prior to inclusion. The protocol was reviewed and approved by the Medical Ethics Committee of Nanjing Stomatological Hospital and the study conforms to the declaration of Helsinki. To quantitatively investigate the amino acids concentration of OSCC samples, frozen tumor tissues from 20 primary OSCC patients were prospectively collected (**Table 1**). There were 13 men and 7 women who had ages ranging from 37 to 73 years (median, 60.5 years). Within these tumor tissues, 8 were judged as PNI positive (PNI^+^, **Figure 1**) by two independent oral pathologists with Hematoxylin and Eosin (H&E) staining.

**Table 1:** Baseline characteristics of 20 prospectively collected OSCC patients.

Patient ID	Gender	Age	Tumor site	Differentiation	pT	pN	PNI
#01	Male	56	Floor of mouth	Well	T3	N1	No
#02	Female	37	Tongue	Well	T3	N0	No
#03	Male	73	Tongue	Well	T2	N1	No
#04	Female	64	Tongue	Poor	T2	N0	No
#05	Female	60	Buccal mucosa	Well	T2	N1	No
#06	Male	63	Floor of mouth	Well	T3	N0	No
#07	Female	62	Buccal mucosa	Well	T4	N0	No
#08	Male	70	Hard Palate	Moderately	T3	N0	No
#09	Male	48	Buccal mucosa	Well	T3	N0	No
#10	Male	71	Gingiva	Well	T4	N0	No
#11	Female	55	Tongue	Well	T2	N0	No
#12	Female	71	Buccal mucosa	Well	T2	N0	No
#13	Male	50	Floor of mouth	Moderately	T4	N1	Yes
#14	Male	61	Tongue	Well	T2	N1	Yes
#15	Male	48	Tongue	Well	T2	N1	Yes
#16	Female	61	Gingiva	Well	T4	N1	Yes
#17	Male	40	Tongue	Well	T2	N0	Yes
#18	Male	68	Tongue	Well	T3	N2	Yes
#19	Male	52	Tongue	Well	T2	N0	Yes
#20	Male	54	Tongue	Moderately	T4	N2	Yes

pT, pathologic T stage; pN, pathologic N stage; PNI, perineural invasion; OSCC, oral squamous cell carcinoma.

To retrospectively evaluate ASNS expression, we conducted a cohort including 86 primary OSCC patients. All patients with complete follow-up information were admitted at Nanjing Stomatological Hospital during 2013 to 2014 and diagnosed with primary OSCC by experienced pathologists from the Department of Pathology (**Table 2**). There were 52 men and 34 women included with ages ranging from 28 to 83 yeas (median, 55 years). The follow-up time ranged from 4 to 79 months (median, 71 months). Patients who had received chemotherapy or radiation therapy before surgery were excluded. All OSCC samples were evaluated according to the WHO classification and the UICC tumor–node–metastasis (TNM) staging system, for which “T” describes the extent of primary tumor (T), “N” refers to absence or presence and extent of overt regional lymph node(s), and “M” depicts the absence or presence of distant metastasis.

**Table 2:** ASNS expression and baseline characteristics of 86 primary OSCC patients.

Characteristics	Total N=86	ASNS expression		χ^2^	*P*
		Low, n (%)	High, n (%)		
Age				0.247	0.619
<60	39 (45.3%)	22 (56.4%)	17 (43.6%)		
≥60	47 (54.7%)	29 (61.7%)	18 (38.3%)		
Gender				0.943	0.332
Female	34 (39.5%)	18 (52.9%)	16 (47.1%)		
Male	52 (60.5%)	33 (63.5%)	19 (36.5%)		
Site				3.58	0.472
Tongue	33 (38.4%)	18 (54.5%)	15 (45.5%)		
Gingiva	18 (20.9%)	10 (55.6%)	8 (44.4%)		
Buccal mucosa	17 (19.8%)	11 (64.7%)	6 (35.3%)		
Floor of mouth	10 (11.6%)	5 (50.0%)	5 (50.0%)		
Others	8 (9.3%)	7 (87.5%)	1 (12.5%)		
T				0.032	0.859
T1 + T2	73 (84.9%)	43 (58.9%)	30 (41.1%)		
T3 + T4	13 (15.1%)	8 (61.5%)	5 (38.5%)		
N				6.32	**0.012**
N-	53 (61.6%)	37 (69.8%)	16 (30.2%)		
N+	33 (38.4%)	14 (42.4%)	19 (57.6%)		
Stage				4.316	**0.038**
I + II	46 (53.5%)	32 (69.6%)	14 (30.4%)		
III + IV	40 (46.5%)	19 (47.5%)	21 (52.5%)		
Grade				1.105	0.293
Well	25 (29.1%)	17 (68.0%)	8 (32.0%)		
Moderately + Poor	61 (70.9%)	34 (55.7%)	27 (44.3%)		
PNI				21.395	**<0.001**
No	38 (44.2%)	33 (86.8%)	5 (13.2%)		
Yes	48 (55.8%)	18 (37.5%)	30 (62.5%)		
Radiation therapy				1.489	0.222
Without	58 (67.4%)	37 (63.8%)	21(36.2%)		
With	28 (32.6%)	14 (50.0%)	14(50.0%)		
Chemotherapy				0.884	0.347
Without	78 (90.7%)	48 (61.5%)	30 (38.5%)		
With	8 (9.3%)	3 (37.5%)	5 (62.5%)		

Chi-square test was used between each baseline characteristic and ASNS expression and P < 0.05 in bold was viewed as significant. −, negative; +, positive; PNI, perineural invasion; ASNS, asparagine synthetase; OSCC, oral squamous cell carcinoma.

Another cohort with 279 OSCC patients from The Cancer Genome Atlas (TCGA) was also conducted (**Supplementary Table 1**). Phenotype and survival data retrieved from the TCGA-HNSC dataset were downloaded from the UCSC Xena website (https://xenabrowser.net/). There were 187 men and 92 women with ages ranging from 19 to 90 years (median, 61 years). The follow-up time was from 11 to 5480 days (median, 641 days). H&E-stained images from the Cancer Digital Slide Archive website (https://cancer.digitalslidearchive.org/) were reviewed for their PNI statuses. Additionally, RNA-seq data (FPKM-UQ) of ASNS were downloaded and the cutoff value 17.72 (the third quantile) was selected in order to separate these patients into ASNS expression low (<17.72, ASNS^low^) or high (≥17.72, ASNS^high^).

### Ultra-High-Performance Liquid Chromatography-Tandem Mass Spectrometer Targeted Quantitative Analysis

The specific operation steps for UHPLC-MS/MS could refer to our previous work (20). Shortly described as follows: Equipped with a Waters ACQUITY UPLC BEH Amide column (100 × 2.1 mm, 1.7 μm; Waters Corporation, USA), an Agilent 1290 Infinity II series UHPLC system (Agilent Technologies, California, USA) was used for the UHPLC separation. Mobile phase A and B were respectively made up of 1% formic acid in water and 1% formic acid in acetonitrile. The column temperature was set to 35°C while the auto-sampler temperature was set to 4°C. For assay development, Agilent 6460 Triple Quadrupole mass spectrometer was connected with an Agilent Jet Stream electrospray ionization interface (Agilent Technologies, California, USA).

Isotope standards used for the quantifications were applied and the optimal Multiple Reaction Monitoring (MRM) parameters of the target metabolites were obtained. Agilent MassHunter Work Station Software (B.08.00, Agilent Technologies, California, USA) was used for the MRM data acquisition and processing. The raw data of amino acids concentration in PNI was in the **Supplementary Table 2**.

### Immunohistochemistry

IHC of formalin fixed paraffin-embedded tissues was performed as previously described (21). ASNS rabbit polyclonal antibody (Sigma-Aldrich Cat# HPA029318, RRID: AB_10602389) was used with the dilution ratio 1:200. The intensity of ASNS immunoreaction was scored as follows: 0 = absence of stained cells; 1 = weak staining; 2 = moderate staining; and 3 = strong staining. The percentage of stained cells was scored as follows: 0 = 0–5% stained cells; 1 = 6–33% stained cells; 2 = 34–66% stained cells; 3 = 67–100% stained cells. ASNS immunoreaction index was calculated by multiplying the staining intensity and the percentage of stained cells. Then, OSCC tissues were divided into low or high group: score = 0–4, ASNS low (ASNS^low^); score = 6–9, ASNS high (ASNS^high^).

### Cell Culture and Reagents

The human OSCC cell lines Cal27, HSC3, CAL33, SCC9, SCC131 and immortalized human oral keratinocyte (HOK) were kept in our lab and were maintained in the Dulbecco’s Modified Eagle Medium, high glucose (DMEM-H) supplemented with 10% fetal bovine serum and 1% penicillin-streptomycin. All cell lines were authenticated using Short Tandem Repeat (STR) analysis and cultured at 37 °C in a standard humidified atmosphere of 5% CO2. All cell culture reagents were obtained from Gibco (ThermoFisher, USA)

### Western Blotting

After cells in six-well plate reached a confluency of 80% to 90%, cells were washed three times with ice-cold PBS and then lysed on ice with SDS lysis buffer (Beyotime, China). Equal amounts of protein lysates (25 μg per lane) were separated by 4% to 12% gradient SDS-polyacrylamide gels (GenScript, USA) for 40 min at 200 V and then transferred onto polyvinylidene difluoride (PVDF) membranes (Millipore, USA) using a wet transfer system (Bio-Rad, USA). Membranes were blocked with 5% bovine serum albumin for 1 h at room temperature. Then PVDF membranes were probed with β-Actin mouse monoclonal antibody (dilution ration 1:10000, Proteintech Cat# 66009-1-Ig, RRID: AB_2687938) and ASNS rabbit polyclonal antibody (dilution ration 1:500, Proteintech Cat# 14681-1-AP, RRID: AB_2060119) at 4°C overnight with gentle shaking. After incubation, PVDF membranes were washed three times with PBST (0.05% Tween20 in PBS) and detected with secondary antibodies conjugated with horseradish peroxides (dilution ratio 1:20000, Invitrogen, USA). Images were captured using Tanon-5200 Chemiluminescent Imaging System (Tanon, China).

### Reverse Transcription and Quantitative Real-Time PCR

Total RNA was extracted from cells using TRIzol reagent (Invitrogen, USA) following the manufacturer’s instructions. The concentration and purity of the RNA were determined by measuring the absorbance at 260 nm and 280 nm using NanoDrop One (ThermoFisher, USA). Total RNA (1 μg) was reverse transcribed in a 20 μL system using HiScript III RT SuperMix (Vazyme, China). Subsequently, qRT‐PCR was performed using ChamQ SYBR qPCR Master Mix (Vazyme, China) and LightCycler 96 (Roche, Switzerland). The primer sequences used were as follows: forward primer 5-GGAAGACAGCCCCGATTTACT-3 and reverse primer 5-AGCACGAACTGTTGTAATGTCA-3 for human ASNS; forward primer 5-CATGTACGTTGCTATCCAGGC-3 and reverse primer 5-CTCCTTAATGTCACGCACGAT-3 for human β-Actin. All primer sequences were purchased from Invitrogen (USA).

### *In Vivo* Perineural Invasion Mouse Model

All of the procedures with animal subjects were approved by the Institutional Animal Care and Use Committee at Medical School of Nanjing University. In this study, 6-week-old male BALB/c nu/nu mice were used. The surgical procedure and cancer cells injection in detail were previously described (22). Briefly, mice were anesthetized and maintained with isoflurane and a 1 cm incision with small scissors was made on the hind limb of the injection side. Then, sciatic nerves were exposed and injected with cancer cells (3x10^4^ in 3 μL PBS) or the same volume PBS with a 10 μL syringe (Hamilton, USA). Put the nerve back and close the skin with 5-0 Nylon sutures. Mice were closely watched for its recovery from anesthesia and wound healing. Sciatic nerve function was measured every three days as previously described (23). The sciatic function index indicates the distance between the first and fifth toes of the mouse hind limbs. In the *in vivo* model, disease progression was recorded when the mouse hind limb became complete nerve paralysis. At the end of the experiment, mice were sacrificed and sciatic nerves and tumor tissues were isolated, measured, and fixed for histological analysis.

### Statistical Analysis

For the identification of significant differentially expressed amino acids in UHPLC-MS/MS, the MetaboAnalyst method was applied with a *P*-value threshold of 0.05 and fold-change (FC) threshold of 1.5. For the heatmap, the clustering distances in X-axes and Y-axes were “Correlation” and “Euclidean” respectively, and the clustering method was “Complete”. The clustering distances in X-axes and Y-axes were “Correlation” and “Euclidean” respectively, and the clustering method was “Complete”. To compare how well each amino acid can distinguish between PNI^+^ and PNI^−^ statuses, receiver operating characteristics (ROC) curves with the Area Under the Curve (AUC) value were drawn. Statistical significance of ASNS IHC staining between PNI^+^ and PNI^−^ samples was determined using unpaired two-tailed Student’s *t*-test. Quantitative analysis of distance between the 1st and 5th toes of mice was determined using pairwise two-tailed Student’s t-test. For comparison of the migration distance in nerve environment between HSC3 and CAL33, P-value was derived from an unpaired two-tailed Student’s t-test. Bar graphs represent as the mean ± the standard error of the mean (s.e.m.).

Survival curves were calculated using the Kaplan–Meier method and compared using the log-rank test. Overall survival (OS) was defined as the time from surgery to death from any cause while disease-specific survival (DSS) was defined as the time from surgery to OSCC caused death. On the other hand, progression-free survival (PFS) was defined as the time from surgery to the time evidence of recurrent or progressive disease was obtained based on clinical diagnosis of recurrence or confirmation of recurrence using imaging, or in instances where patients died from OSCC prior to the censoring date.

## Results

### l-Asparagine Provides the Optimal Diagnostic Efficacy for Perineural Invasion

**Figure 2:**
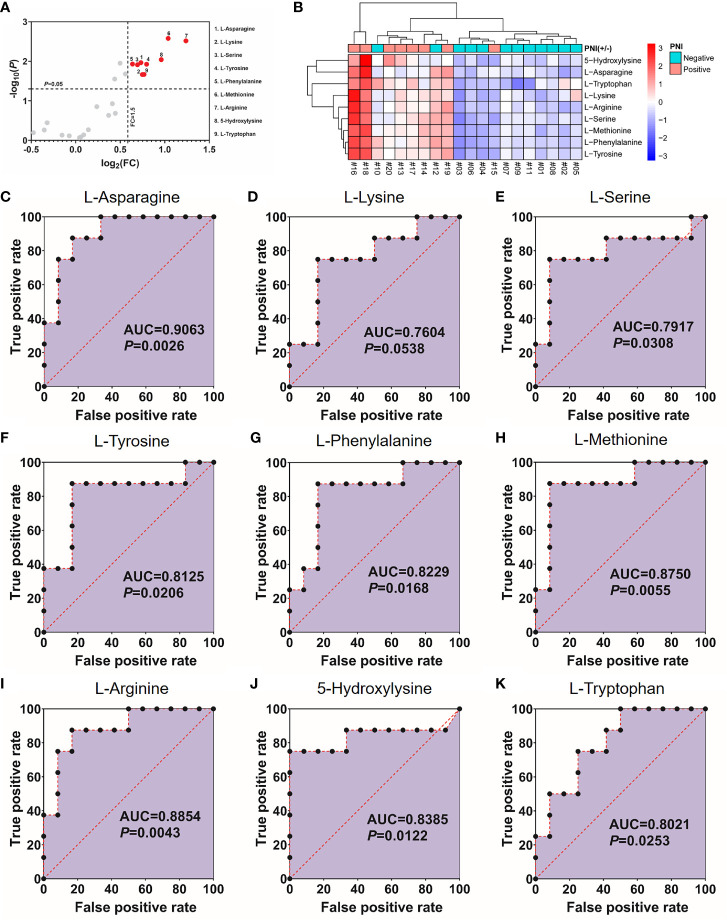
Nine amino acids significantly up-regulated in the PNI^+^ tissues. **(A)** Volcano plot with fold change (FC) threshold = 1.5 and *P* value threshold = 0.05. The red dots represent features above the threshold. Note that both FC and *P* values are log transformed. **(B)** Heatmap visualization of the differential amino acids between samples with different PNI statuses. Automatic clustering by samples (X-axes) and amino acids (Y-axes). The clustering distances in X-axes and Y-axes were “Correlation” and “Euclidean” respectively, and the clustering method was “Complete”. **(C–K)** Receiver operating characteristics curves for each of the nine amino acids above. FC, fold change; AUC, area under the curve; PNI, perineural invasion; OSCC, oral squamous cell carcinoma. +, positive; −, negative.

As shown in **Figure 1**, 40% (8/20) prospectively collected OSCC samples were diagnosed with PNI^+^. Targeted quantitative metabolomics analyses were performed for 48 amino acids using UHPLC-MS/MS and 25 amino acids were detected robustly, among which 9 significantly up-regulated in the PNI^+^ samples (**Figures 2A, B**). The ability of single amino acid marker distinguishing the PNI^+^ samples from the PNI negative (PNI^−^) was tested using ROC curve analysis. With the results shown that only one amino acid marker (l-asparagine, AUC > 0.9) displayed high sensitivity and specificity in diagnosing the PNI (**Figure 2C**). In addition, two amino acid markers displayed the least sensitivity and specificity (AUC < 0.8, **Figures 2D, E**) and six displayed moderate sensitivity and specificity (AUC = 0.8–0.9, **Figures 2F-K**). Since its well-known role in the patients with acute lymphoblastic leukemia, the new discovery of l-asparagine dysregulation behind PNI deserves further exploration (24).

### High Expression of Asparagine Synthetase in Tumor Cells Positively Correlated With Perineural Invasion

**Figure 3:**
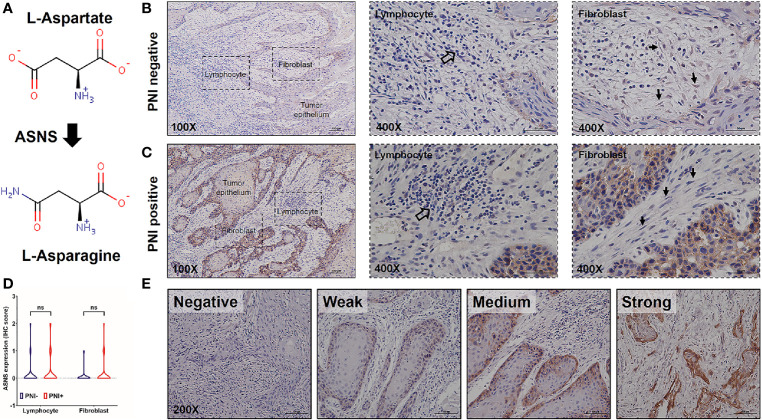
The expression pattern of ASNS in the OSCC tissues. **(A)** ASNS catalyzed the synthesis of l-asparagine from l-aspartate. **(B, C)** Representative IHC images of ASNS separately in the PNI^+^ **(B)** and PNI^−^ **(C)** tissues. Note that the open arrows indicate the lymphocytes and solid arrows indicate the fibroblasts. **(D)** Comparison of ASNS expression in the lymphocytes (or fibroblasts) between the PNI^+^ and PNI^−^ OSCC tissues. Statistical significance of ASNS IHC staining between PNI^+^ and PNI^−^ samples was determined using unpaired two-tailed Student’s *t*-test. **(E)** Illustration of the intensity of ASNS expression in the tumor epitheliums. ASNS, asparagine synthetase; IHC, immunohistochemistry; PNI, perineural invasion; OSCC, oral squamous cell carcinoma. ns, not statistically significant (*P* ≥ 0.05). +, positive; −, negative.

Since ASNS is the key enzyme for l-asparagine metabolism (**Figure 3A**), ASNS expression in OSCC samples was evaluated by IHC staining. As shown in **Figures 3B, C**, ASNS was mainly located in the tumor epithelium while there was low or no expression in the lymphocytes and fibroblasts. With regards to the ASNS expression in the lymphocytes and fibroblasts, there was no significant difference between the PNI^+^ and PNI^−^ samples (**Figure 3D**). However, the intensity of ASNS expression in the tumor epithelium varied apparently among individuals (**Figure 3E**).

**Figure 4:**
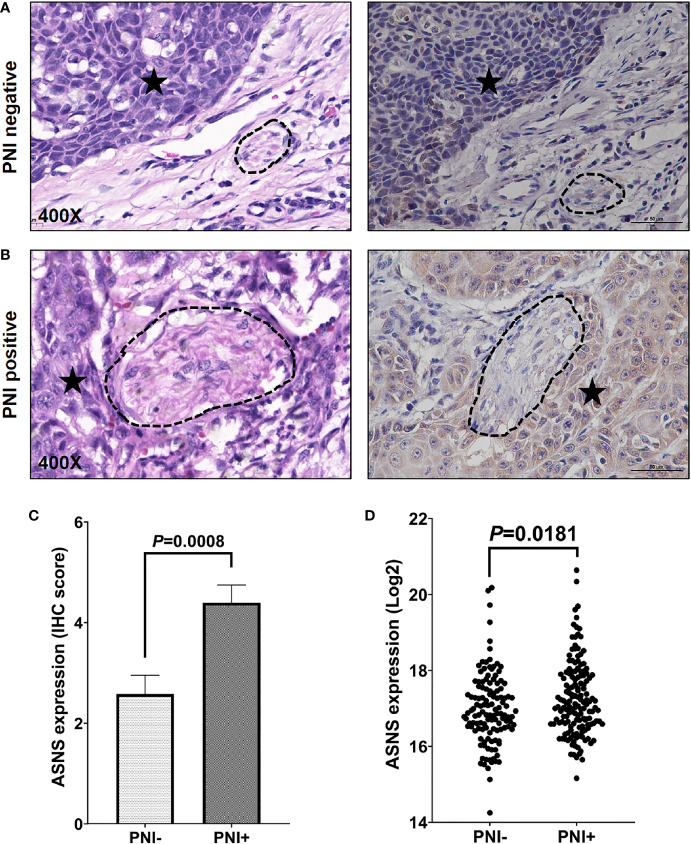
ASNS expression significantly upregulated in the PNI^+^ OSCC tissues. **(A, B)** Comparison of ASNS expression in the tumor epitheliums of the PNI^+^ **(B)** and PNI^−^ **(A)** tumors by the IHC staining (Right panel). The left panel shows the same tumor area as the right panel by the H&E staining and the dark asterisk indicates the tumor while the dashed circle shows the nerve trunk. **(C)** The IHC scores of ASNS expression between the PNI^+^ and PNI^−^ tumors. Data are presented as mean ± s.e.m. **(D)** Comparison of the ASNS mRNA expression from the TCGA between the PNI^+^ and PNI^−^ tumors. Statistical significance of ASNS expression between PNI^+^ and PNI^−^ samples was determined using unpaired two-tailed Student’s *t*-test. ASNS, asparagine synthetase; IHC, immunohistochemistry; H&E, hematoxylin eosin; PNI, perineural invasion; OSCC, oral squamous cell carcinoma. +, positive; −, negative.

The clinicopathological data in **Table 2** indicated that ASNS expression was positively correlated with the pathologic N stage (χ^2^ = 6.32, *P* = 0.012), TNM stage (χ^2^ = 4.316, *P* = 0.038), and PNI (χ^2^ = 21.395, *P* < 0.001), but not T stage (χ^2^ = 0.032, *P* = 0.859). Obtained IHC images showed that PNI^+^ OSCC samples had an upregulated ASNS expression in the tumor epithelium (**Figures 4A, B**) with significantly increased IHC score (**Figure 4C**, *P* = 0.0008). TCGA data confirmed our results that PNI^+^ OSCCs had significantly elevated ASNS expression at the mRNA level (**Figure 4D**).

### The PNI^+^ASNS^high^ Oral Squamous Cell Carcinoma Patients Had the Worst Survival Outcome

**Figure 5:**
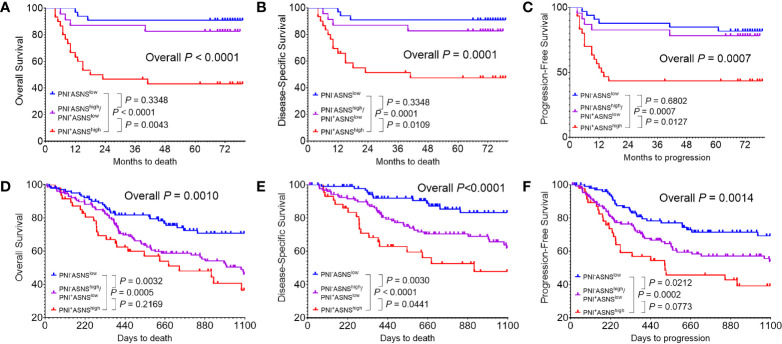
Evaluation of the prognostic value of combinations of the PNI status and ASNS expression **(A–C)** Kaplan-Meier analysis in the 86 OSCC patients cohort from our hospital for the overall survival **(A)**, disease-specific survival **(B)**, and progression-free survival **(C)**. **(D–F)** Survival analysis in the 279 OSCC patients cohort from the TCGA. Survival curves were calculated using the Kaplan–Meier method and compared using the log-rank test. ASNS, asparagine synthetase; PNI, perineural invasion; OSCC, oral squamous cell carcinoma. +, positive; −, negative.

Regarding that PNI positively correlated with ASNS expression, OSCC patients were classified into three groups: I, PNI^−^ASNS^low^; II, PNI^−^ASNS^high^/PNI^+^ASNS^low^; III, PNI^+^ASNS^high^. Kaplan-Meier analysis revealed that patients in group III had the worst OS (*P* < 0.0001, **Figure 5A**), DSS (*P* = 0.0001, **Figure 5B**), and PFS (*P* = 0.0007, **Figure 5C**). TCGA data with larger population indicated that combinations of the PNI status and ASNS expression robustly distinguished three groups of patients with varied prognosis (**Figures 5D–F**). In detail, comparative to the patients in group II, individuals in group I always had significantly better OS (*P* = 0.0032), DSS (*P* = 0.0030), and PFS (*P* = 0.0212), while ones in group III had relatively worse OS (*P* = 0.2169), DSS (*P* = 0.0441), and PFS (*P* = 0.0773).

### Asparagine Synthetase Promoted Nerve Invasion of Oral Squamous Cell Carcinoma Cells in the Mouse Perineural Invasion Model *In Vivo*

**Figure 6:**
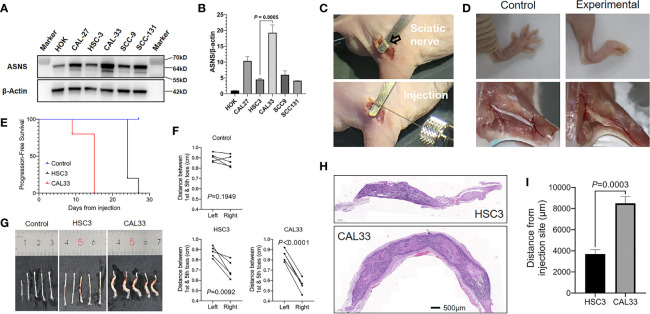
ASNS promotes cancer cells neve invasion *in vivo* **(A, B)** ASNS levels in several cell lines were screened by the western blotting **(A)** and the quantitative real-time PCR **(B)**. **(C)** Exposure of the sciatic nerve (open arrow) and injection. **(D)** Comparison of the nerve functions (upper panel) and appearances (lower panel) between the control mice (left panel, injected with PBS) and experimental mice (right panel, injected with cancer cells). **(E)** Time to nerve dysfunction among three groups of mice (Control, HSC3 and CAL33) were compared. **(F)** The distance between the 1st and 5th toes of mice was recorded to represent the sciatic function index. Quantitative analysis of distance between the 1st and 5th toes of mice was determined using pairwise two-tailed Student’s *t*-test. **(G)** Images showing the sciatic nerves of the three groups of mice. **(H)** Representative H&E-stained images of the cancer cells in the sciatic nerves (HSC3, upper panel; CAL33, lower panel). **(I)** Quantitative comparison of the distance of cancer cells (HSC3 vs. CAL33) migration from the injection sites. Five mice were used in each *in vivo* model. For comparison of the migration distance in nerve environment between HSC3 and CAL33, *P*-value was derived from an unpaired two-tailed Student’s *t*-test. Bar graphs represent as the mean ± the standard error of the mean (s.e.m.) ASNS, asparagine synthetase.

We next compared ASNS expression levels across 6 cell lines. As shown in **Figures 6A, B**, when compared to immortalized human oral keratinocyte (HOK), OSCC cell lines (CAL27, HSC3, CAL33, SCC9, and SCC131) had significantly higher expression of ASNS. Among these five cancer cell lines, we finally chose HSC3 and CAL33 to construct the *in vivo* PNI mouse model as HSC3 had the lowest ASNS expression while CAL33 had the highest. As demonstrated in **Figure 6C**, with cancer cells or PBS injection into the sciatic nerves, mice eventually developed the hind-limb paralysis or kept normal (**Figure 6D**, upper panel). What is more, comparative to the control mice (injection with PBS), the experimental mice successfully developed tumors in the sciatic nerves (**Figure 6D**, lower panel).

With the *in vivo* PNI mouse models successfully constructed, we subsequently compared the sciatic function index among three groups of mice: the control group with PBS injection, the HSC3 group with HSC3 injection, and the CAL33 group with CAL33 injection. As shown in **Figure 6E**, the control group did not show any nerve dysfunctions during the entire experimental period while 4/5 mice in the HSC3 group developed nerve dysfunction at Day 24. Importantly, in the CAL33 group 1/5 mice became hind-limb paralysis at Day 9 and all mice developed nerve dysfunction at Day 15. Distances between the first and fifth toes confirmed that mice in the CAL33 group eventually had more severe sciatic nerves dysfunction than the HSC3 group (*P* < 0.0001 vs. *P* = 0.0092, **Figure 6F**). As demonstrated in **Figure 6G**, tumor burden in the CAL33 group was larger than that in HSC3 group. H&E-stained images of the injected nerves (long axis) revealed that cancer cells invasion distance in the nerve microenvironment varied between the HSC3 and CAL33 groups (**Figure 6H**). Quantification results indicated that CAL33 had significantly longer nerve invasion distance than HSC3 (*P* = 0.0003, **Figure 6I**).

## Discussion

Although a combination of non-targeted and targeted metabolomics have revealed the aberrant levels of several amino acids from normal epithelium to OSCC, the potential function of dysregulated amino acids metabolism behind OSCC metastasis is still unclear (17). PNI, as a third route for tumor dissemination besides the local invasion and lymphovascular metastasis, was demonstrated in this study to have correlations with the amino acids metabolism disorder. We found that l-asparagine was significantly enriched in the PNI^+^ samples and its key enzyme ASNS overexpression accelerated nerve dysfunction and promoted cancer cells invasion in the nerve microenvironment.

PNI as a well-known pathological feature has been recognized widely as an indicator of poor prognosis and we here investigated the metabolism change between PNI^+^ and PNI^−^ samples, thus two groups were divided in first parts of this study, whereas the amino acid l-asparagine and its key enzyme ASNS was found to be significantly correlated with PNI statuses, and three groups was divided based on both PNI status and ASNS expression in further study to emphasize the potential role of ASNS during the PNI development.

Although there were total 9 amino acids displaying upregulated in the PNI^+^ OSCC samples, we finally chose l-asparagine as the marker to distinguish different PNI statuses. As one of the non-essential amino acids (NEAAs), l-asparagine strongly influences tumor metastatic potential (25, 26). Supplementing the culture medium with l-asparagine increased the invasiveness of breast cancer cells twofold when compared with other NEAAs (25). Moreover, concentrations of l-asparagine gradually increased with progression from normal oral tissues to OSCC (20). As illustrated in **Figure 2B**, the expressions of amino acids in patients #10, #12, and #15 were indeed the outliers, indicating the existence of individual differences. One possible explanation is that individual factors such as drug history, dietary habit et al. may affect their amino acids metabolism levels and the other possibility may be due to the limited sample size (n = 20) of this study. Although we believed that these three cases should not be deleted, the AUC value after deletion confirmed that l-asparagine still had an optimal diagnostic efficacy (AUC = 0.9571, *P* = 0.0018) for PNI, suggesting that elevated l-asparagine level promoted OSCC progression through enhanced PNI formation.

ASNS regulates the metabolism level of l-asparagine. To verify that whether ASNS associated with PNI the same pattern as its substrate l-asparagine, we investigated the relationship between the clinicopathological data of OSCC patients and ASNS expression at the protein or mRNA level. Consistently, ASNS expression was positively correlated with PNI and negatively with the survival outcome. Recently, a phase IIb open-label study evaluated the effect of l-asparaginase combined with chemotherapy in the second-line treatment of advanced pancreatic ductal adenocarcinomas (PDACs) showing that the combination was associated with improvements in OS and PFS (27). According to the human protein atlas data (HPA, https://www.proteinatlas.org/ENSG00000070669-ASNS/tissue), ASNS expression was high in the normal pancreas but low in the normal oral mucosa. In cancer tissues, high ASNS expression was found in only 20% to 50% of the resected PDACs (28, 29). Similarly, in this study of the resected OSCCs 41% (35/86) at the protein level and 25% (71/279) at the mRNA level showed high expression of ASNS. Considering the similar expression pattern as the PDAC, l-asparaginase has excellent therapeutic potential for OSCC treatment.

The mechanism of ASNS promoting OSCC nerve invasion still needs to be further explored. In order to construct the *in vivo* PNI mouse models using oral cancer cells with different ASNS baseline expressions, we screened 5 OSCC cell lines and 1 normal human oral keratinocyte cell line in our lab. Consistent with our previous work, cancer cells had significantly elevated ASNS expression than the normal cell (HOK) but varied distinctly among OSCC cell lines (20). Besides selecting two OSCC cell lines (HSC3 and CAL33) with different ASNS levels, knockdown or overexpression of ASNS in cancer cells through genetic modification was another reliable way to construct the *in vivo* PNI model. Although we could not explain at present through which signaling pathway ASNS promotes PNI, the amino acids metabolism alterations behind the nerve invasion of OSCC need be noticed. As reported, the activating transcription factor (ATF)-4 targeted ASNS and knockdown of ATF-4 significantly reduced ASNS expression (30–32). Moreover, induction of ATF-4 is dependent on the activation of the PI3K-AKT-mTOR signaling (31, 33). Interestingly, PNI^+^ tumors had increased activation levels of the AKT and mTOR kinases (34). Therefore, ASNS regulating PNI may be involved in the PI3K-AKT-mTOR-ATF4 signaling pathway.

In conclusion, l-asparagine and its key enzyme ASNS are involved in the process of PNI which is validated based on the clinicopathological data and an *in vivo* PNI mouse model. Elucidation of ASNS-mediated l-asparagine metabolism alteration behind PNI in the future would provide novel therapeutic targets for inhibiting OSCC early dissemination.

## Data Availability Statement

Publicly available datasets were analyzed in this study. These data can be found here: https://gdc.xenahubs.net/download/TCGA-HNSC.htseq_fpkm-uq.tsv.gz.

## Ethics Statement

The studies involving human participants were reviewed and approved by the Research Ethics Committee of Nanjing Stomatological Hospital. The patients/participants provided their written informed consent to participate in this study. The animal study was reviewed and approved by the Institutional Animal Care and Use Committee at Medical School of Nanjing University.

## Author Contributions

QH and YN designed this study. YF and LD performed all the experiments. YF, LD, and ZD collected the clinical data. YF, LD, and XY interpreted the data. XH, LZ, and SC offered technical support. YF and LD wrote the manuscript. All authors contributed to the article and approved the submitted version.

## Funding

This work was supported by the National Natural Science Foundation of China (grant nos. 81902754, 81772880, 81700939, and 81902759), Fundamental Research Funds for the Central Universities (no. 021014380161), Natural Science Foundation of Jiangsu Province (no. BK20190304), China Postdoctoral Science Foundation (no. 2019M651789), and Nanjing Medical Science and Technology Development Foundation, Nanjing Department of Health (no. YKK18123).

## Conflict of Interest

The authors declare that the research was conducted in the absence of any commercial or financial relationships that could be construed as a potential conflict of interest.
