# Supplementary material for: Asparagine Synthetase-Mediated l-Asparagine Metabolism Disorder Promotes the Perineural Invasion of Oral Squamous Cell Carcinoma
Source: Front Oncol. 2021 Mar 10;11:637226. doi: 10.3389/fonc.2021.637226 (PMC7987891; doi:10.3389/fonc.2021.637226)
Supplement: Supplementary file 1 [file Table_1.docx]

Supplementary Material

**Supplementary Table 1.** ASNS expression and baseline characteristics of 279 OSCC patients from the TCGA

| **Characteristics** | **Total**  **N=279** | **ASNS expression** | | **χ^2^** | ***P*** |
| --- | --- | --- | --- | --- | --- |
|  |  | **Low, n (%)** | **High, n (%)** |  |  |
| Age |  |  |  | 0.117 | 0.733 |
| <60 | 117 (41.9%) | 86 (73.5%) | 31 (26.5%) |  |  |
| ≥60 | 162 (58.1%) | 122 (75.3%) | 40 (24.7%) |  |  |
| Gender |  |  |  | 0.995 | 0.318 |
| Female | 92 (33.0%) | 72 (78.3%) | 20 (21.7%) |  |  |
| Male | 187 (67.0%) | 136 (72.7%) | 51 (27.3%) |  |  |
| Site |  |  |  | 6.05 | 0.190 |
| Tongue | 112 (40.1%) | 89 (79.5%) | 23 (20.5%) |  |  |
| Gingiva | 17 (6.1%) | 12 (70.6%) | 5 (29.4%) |  |  |
| Buccal mucosa | 21 (7.5%) | 13 (61.9%) | 8 (38.1%) |  |  |
| Floor of mouth | 56 (20.1%) | 37 (66.1%) | 19 (33.9%) |  |  |
| Others | 73 (26.2) | 57 (78.1%) | 16 (21.9%) |  |  |
| T |  |  |  | 0.047 | 0.829 |
| T1 + T2 | 117 (41.9%) | 88 (75.2%) | 29 (24.8%) |  |  |
| T3 + T4 | 162 (58.1%) | 120 (74.1%) | 42 (25.9%) |  |  |
| N |  |  |  | 2.588 | 0.108 |
| N- | 133 (47.7%) | 105 (78.9%) | 28 (21.1%) |  |  |
| N+ | 146 (52.3%) | 103 (70.5%) | 43 (29.5%) |  |  |
| Stage |  |  |  | 1.119 | 0.290 |
| I + II | 68 (24.4%) | 54 (79.4%) | 14 (20.6%) |  |  |
| III + IV | 211 (75.6%) | 154 (73.0%) | 57 (27.0%) |  |  |
| Grade |  |  |  | 2.391 | 0.303 |
| G1 | 45 (16.1%) | 35 (77.8%) | 10 (22.2%) |  |  |
| G2 | 177 (63.4%) | 135 (76.3%) | 42 (23.7%) |  |  |
| G3 | 57 (20.4%) | 38 (66.7%) | 19 (33.3%) |  |  |
| PNI |  |  |  | 4.961 | **0.026** |
| No | 126 (45.2%) | 102 (81.0%) | 24 (19.0%) |  |  |
| Yes | 153 (54.8%) | 106 (69.3%) | 47 (30.7%) |  |  |
| Radiation therapy |  |  |  | 5.08 | **0.024** |
| Without | 99 (35.5%) | 82 (82.8%) | 17 (17.2%) |  |  |
| With | 144 (51.6%) | 101 (70.1%) | 43 (29.9%) |  |  |
| NA | 36 (12.9%) | 25 (69.4%) | 11 (30.6%) |  |  |
| Target therapy |  |  |  | 0.466 | 0.495 |
| Without | 163 (58.4%) | 126 (77.3%) | 37 (22.7%) |  |  |
| With | 59 (21.1%) | 43 (72.9%) | 16 (27.1%) |  |  |
| NA | 57 (20.4%) | 39 (68.4%) | 18 (31.6%) |  |  |

Notes: Data with NA was not included in the Chi-square test and *P* < 0.05 in bold was viewed as significant. Abbreviations: -, negative; +, positive; NA, not available; PNI, perineural invasion; ASNS, asparagine synthetase; OSCC, oral squamous cell carcinoma
